# Real-time torque behavior of reciprocating nickel–titanium instruments using different irrigating solutions

**DOI:** 10.1371/journal.pone.0347424

**Published:** 2026-04-17

**Authors:** Uğur Dursun, Mevlüt Sinan Ocak

**Affiliations:** 1 Batman University, Faculty of Dentistry, Department of Endodontics, Batman, Turkey; 2 Firat University, Faculty of Dentistry, Department of Endodontics, Elazıg, Turkey; Universidade Federal Fluminense, BRAZIL

## Abstract

Reciprocating nickel–titanium instruments generate mechanical stress during root canal preparation, which may affect instrument safety. Although instrument design is known to influence torque generation, the effect of different irrigating solutions on the real-time operative torque remains unclear. This study aimed to investigate the impact of various irrigating solutions on torque generated during root canal shaping using reciprocating single-file systems. Extracted human mandibular premolars with oval canals were assigned to groups prepared using sodium hypochlorite, ethylenediaminetetraacetic acid, or saline in combination with three reciprocating file systems. All canals were instrumented with a torque-controlled motor, and the real-time operative torque and preparation time were digitally recorded. The mean torque, maximum torque, and shaping duration were statistically compared between the irrigant and instrument groups. The irrigation solution alone did not show a significant main effect on torque values or preparation time. However, a significant interaction between the file system and irrigant type was detected, indicating that the torque response depended on the specific file–irrigant combination. In contrast, the file system type significantly influenced torque behavior, with the T-Endo MUST system producing higher mean and peak torque than the other instruments. These findings suggest that instrument design characteristics may have a greater impact on the development of mechanical stress during root canal preparation than the irrigation solution used.

## Introduction

The primary objectives of root canal treatment are to eliminate pulp tissue, microorganisms, and their by-products from the root canal system, and to prevent reinfection. Mechanical instrumentation significantly reduces the microbial load; however, the anatomical complexity of the root canal system often results in substantial unprepared areas that may harbor residual biofilms. Therefore, chemomechanical preparation is essential to enhance canal cleanliness and improve disinfection. Irrigation contributes critically to this process by promoting debris removal, reducing bacterial contamination, and aiding the management of the smear layer on instrumented dentin surfaces [1,2].

The clinical use of rotary nickel–titanium (NiTi) file systems has become increasingly common, as they provide greater flexibility, improved shape maintenance, and enhanced shaping efficiency compared to stainless steel instruments [3]. Consequently, the design characteristics, metallurgy, and thermomechanical treatment of NiTi instruments have been the focus of numerous recent studies [4,5]. Reciprocating single-file systems reduce the number of instruments required, offer faster preparation, and exhibit resistance to cyclic fatigue, thereby decreasing the risk of file fracture and gaining clinical popularity [6].

During root canal shaping, the contact between the instrument and dentin may lead to torsional stress, friction, and the formation of microcracks [7,8]. Torque represents the force applied to rotate NiTi instruments within the canal and is one of the key determinants of the stress generated during mechanical preparation [8]. The amount of torque produced is influenced by several factors, including the canal anatomy, instrument design, kinematics, and operator pressure [9,10]. Clinically, excessive torque values are of critical importance, as they may result in torsional fatigue, sudden file separation, and microstructural damage to dentin [6,11].

Although numerous studies have evaluated cyclic fatigue and torsional resistance under static conditions, these tests do not fully reflect the dynamic mechanical behavior of instruments during clinical use [12]. Real-time torque analysis provides a more clinically relevant assessment by capturing continuous stress fluctuations during instrumentation, allowing a more meaningful comparison of the mechanical performance and safety profiles of different NiTi systems [8,10].

Although the effects of root canal irrigating solutions on dentin surfaces are well established, their influence on operative torque during preparation remains unclear. Current literature indicates that irrigant composition may affect the maximum torque and vertical force values [13], and that different irrigation/lubrication environments can alter real-time operative torque measurements [14]. Sodium hypochlorite (NaOCl) removes the organic components of dentin, ethylenediaminetetraacetic acid (EDTA) dissolves inorganic content, and saline is frequently used as a control solution because of its neutral nature. Therefore, it is important to investigate the potential effects of irrigation solutions on torque production.

Dynamic torque analysis offers a more realistic representation of the mechanical behavior of NiTi alloys than conventional static torsional or cyclic fatigue tests because it captures continuous stress fluctuations under clinically relevant dynamic loading [8,9]. Although the interest in real-time torque monitoring has increased in recent years, few studies have evaluated how different chemical environments alter the torsional response of reciprocating NiTi instruments [13^,^^14^]. In addition, heat-treated NiTi systems such as T-Endo MUST have only recently been introduced, and their torque behavior under different chemical environments has not yet been thoroughly characterized. However, the combined influence of different irrigating solutions and reciprocating kinematics on real-time torque behavior of modern heat-treated NiTi instruments remains insufficiently investigated.

Reciproc Blue, WaveOne Gold, and the more recently introduced T-Endo MUST system differ in metallurgical modifications, cross-sectional designs, and tapers, and operate using a reciprocating motion [15,16]. Examining torque variations between these systems is important for clinical safety considerations [8]. The aim of this study was to evaluate the effects of different irrigating solutions on real-time torque and preparation time during root canal shaping using three reciprocating single-file systems. The null hypotheses tested were as follows: (i) irrigating solutions would not produce statistically significant differences in operative torque parameters or preparation time, and (ii) no significant differences would be found among the reciprocating single-file systems regarding these parameters.

## Materials and methods

This in vitro study was planned and conducted at the Department of Endodontics, Faculty of Dentistry, Fırat University. The study protocol was approved by the Fırat University Non-Interventional Clinical Research Ethics Committee on 16.09.2021 (Decision no: 2021/09–27). Written informed consent was obtained from the patients for the use of their extracted teeth for research purposes. All procedures complied with the principles of the World Medical Association Declaration of Helsinki for medical research involving human tissue samples, ensuring donor anonymity and confidentiality. The recruitment period for this study started on 22/11/2021 and ended on 02/06/2022. The sample size was determined using G*Power 3.1 software, with an effect size (f) of 0.30, a power (1-β) of 0.90, and alpha error threshold of 0.05, resulting in a total of 90 samples (n = 90).

**Fig 1:**
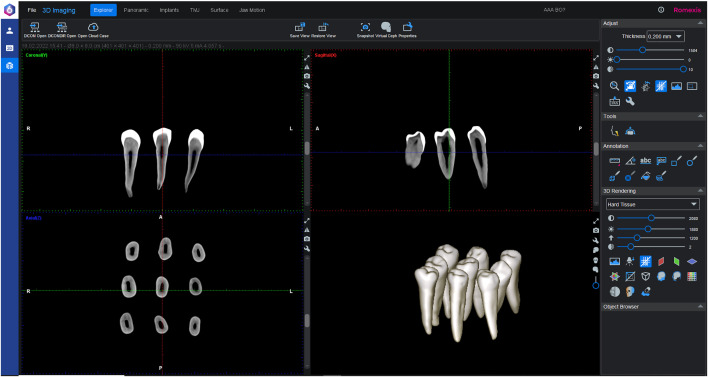
Three-dimensional CBCT reconstructions illustrating oval canal morphology, Vertucci Type I configuration, and curvature < 30° in mandibular premolars selected for this study.

Initially, 167 extracted human mandibular premolars were screened to identify the teeth that met the inclusion criteria. Conventional two-dimensional periapical radiographs and three-dimensional cone-beam computed tomography (CBCT) images were obtained using a Planmeca ProMax 3D Mid unit (Helsinki, Finland; 60–120 kV, 1–14 mA, 200-µm voxel size). Root canal morphology was evaluated using Planmeca Romexis Viewer 6.1 software (Fig 1).

For anatomical standardization, the minimum canal diameter was measured in the mesiodistal direction, and the maximum diameter was measured in the buccolingual direction. Teeth demonstrating a buccolingual–mesiodistal ratio greater than 2:1 were selected to ensure oval canal geometry. Only teeth presenting with Vertucci Type I canal configuration and apical curvature less than 30°, according to the Schneider classification, were included. Following this screening, 90 mandibular premolars meeting all the criteria were assigned to the study.

Teeth presenting with root fractures, resorptions, calcifications, or a history of previous endodontic treatment were excluded. Soft tissue remnants were cleaned and the samples were stored in 0.1% thymol solution until use. The root lengths were standardized to 16 mm by flattening the coronal reference surface. The working length was established by inserting a #10 K-file until its tip was visible at the apical foramen and subtracting by 1 mm.

### Grouping

The samples were randomly distributed into nine experimental groups (n = 10) according to the irrigant type and reciprocating file system.

### Irrigation solutions

- 5% Sodium hypochlorite (NaOCl)
- 17% Ethylenediaminetetraacetic acid (EDTA)
- Physiological saline (control)

### Reciprocating single-file systems

- Reciproc Blue R25 (VDW, Germany)
- WaveOne Gold Primary (Dentsply Sirona, Sweden)
- T-Endo MUST M25 (Dentac, Türkiye)

Total grouping: 3 irrigants × 3 systems = 9 groups.

### Biomechanical preparation and real-time torque recording

All root canals were instrumented by a single experienced operator using a torque-controlled X-Smart IQ endodontic motor, operating in dedicated reciprocation modes recommended by the manufacturers. Real-time torque monitoring was enabled by an integrated recording system that captured torque fluctuations at short intervals during clinical use.

Each file was used to prepare only one canal to eliminate metal fatigue artifacts. Patency was maintained using a #15 K-file throughout instrumentation. All procedures were performed at 37 ± 1 °C to simulate intraoral conditions and to maintain physiological dentin behavior. All instrumentation procedures were performed by a single experienced operator. Although the operator was aware of the instruments used during preparation, torque values were recorded automatically by the digital monitoring system, and the subsequent data processing and statistical analyses were conducted independently to minimize operator-related bias.

During preparation, irrigants facilitated debris removal and file advancement within the canal. A total irrigant volume of 10 ml per canal was used. Irrigation was performed using a 30-gauge side-vented needle positioned 2 mm short of the working length. No ultrasonic or sonic activation was performed.

The real-time operative torque and recording capability of the X-Smart IQ enabled the dynamic evaluation of file behavior and peak stress generation under clinical simulation conditions. The following parameters were extracted digitally:

- Mean operative torque (N·cm)
- Maximum (peak) torque (N·cm)
- Total preparation time (seconds)

Data were exported as Excel spreadsheets for statistical processing.

### Statistical analysis

The data obtained in this study were analyzed using the SPSS 26.0 statistical software package (IBM, Armonk, NY, USA). Prior to the analyses, the normality of data distribution was assessed. The Kolmogorov–Smirnov and Shapiro–Wilk tests revealed p-values greater than 0.05, confirming that the data were normally distributed; therefore, parametric tests were employed. To determine whether different file systems and irrigating solutions caused statistically significant differences in preparation time, mean torque, and maximum torque values, one-way analysis of variance (ANOVA) was applied, followed by Bonferroni post-hoc testing for multiple comparisons. In addition, the interaction effect of the file system and irrigating solution on these parameters was evaluated using two-way ANOVA. Two-way ANOVA was specifically used to evaluate potential interaction effects between file system type and irrigating solution on torque generation and preparation time. Differences were considered statistically significant at p < 0.05 throughout the analysis. The complete dataset used for statistical analysis is provided as Supporting Information (S1 Data).

## Results

### Effect of irrigating solutions

**Fig 2:**
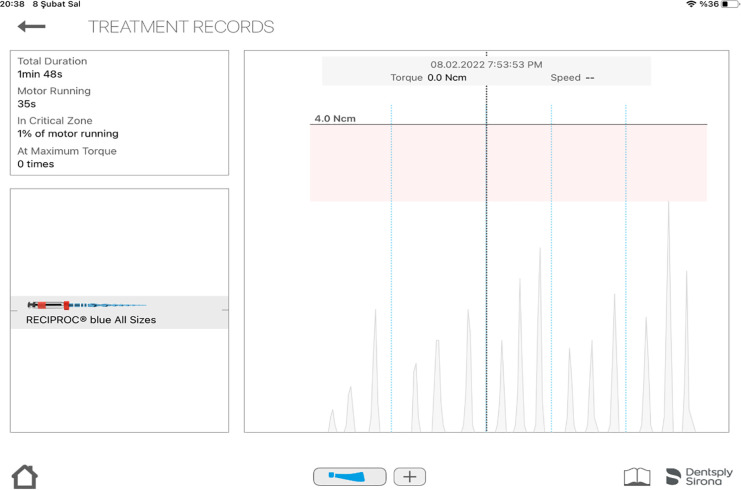
Representative real-time operative torque curve obtained during reciprocating instrumentation, showing continuous torque fluctuations, transient peak values.

No statistically significant differences were observed among the irrigating solutions in terms of the mean operative torque, maximum torque, and preparation time. Although EDTA resulted in slightly shorter preparation times, these differences were not statistically significant (Tables 1 and 2). A representative real-time operative torque curve obtained during reciprocating instrumentation is shown in Fig 2.

**Table 1:** Comparison of Preparation Time by Irrigating Solution (seconds).

Irrigating solution	n	m ± sd	min	max
Saline	30	99.57 ± 22.67	67.00	161.00
Sodium hypochlorite	30	97.37 ± 19.98	65.00	133.00
EDTA	30	92.23 ± 14.74	65.00	133.00
Total	**90**	**96.39 ± 19.43**	65.00	161.00

* One-way ANOVA, p = 0.328. No statistically significant differences were observed between the groups.

**Table 2:** Comparison of Mean and Maximum Torque by Irrigating Solution (N·cm).

	mean				max			
Irrigating solution	n	m ± sd	min	max	n	m ± sd	min	max
Saline	30	1.86 ± 0.61	0.75	3.25	30	3.15 ± 0.86	1.50	4.90
Sodium hypochlorite	30	1.92 ± 0.39	1.31	2.77	30	3.07 ± 0.68	1.80	4.70
EDTA	30	1.96 ± 0.38	1.27	2.88	30	2.95 ± 0.60	1.70	4.30
Total	**90**	**1.91 ± 0.47**	0.75	3.25	90	3.06 ± 0.72	1.50	4.90

*One-way ANOVA, p = 0.705 for mean torque. *One-way ANOVA, p = 0.582 for maximum torque

### Effect of file systems

The file system selection significantly influenced both the mean torque (p = 0.001) and maximum torque values (p = 0.001). T-Endo MUST generated higher torque values than WaveOne Gold and Reciproc Blue (p < 0.05). WaveOne Gold exhibited the lowest torque values. No significant difference was detected between WaveOne Gold and Reciproc Blue (p > 0.05) (Table 3). The preparation time was not significantly affected by file system type (p = 0.216).

**Table 3:** Comparison of Mean and Maximum Torque by File System (N·cm).

	mean				max			
File system	n	m ± sd	min	max	n	m ± sd	min	max
Reciproc Blue	30	1.81 ± 0.47	0.75	2.77	30	3.04 ± 0.77	1.50	4.70
WaveOne Gold	30	1.70 ± 0.29	1.09	2.47	30	2.70 ± 0.42	2.10	3.80
T-Endo MUST	30	2.04 ± 0.45	1.57	3.25	30	3.43 ± 0.74	2.10	4.90
Total	**90**	**1.91 ± 0.47**	0.75	3.25	90	**3.06 ± 0.72**	1.50	4.90

*Note. For the Mean Torque: One-way ANOVA was used, p = 0.001. Post-hoc (Bonferroni): T-Endo MUST > WaveOne Gold and Reciproc Blue (p < 0.05). There was no significant difference between Reciproc Blue and WaveOne Gold (p > 0.05). For the Maximum Torque: One-way ANOVA was used, p = 0.001. Post-hoc (Bonferroni): T-Endo MUST > WaveOne Gold (p < 0.05). Reciproc Blue ~ T-Endo MUST and Reciproc Blue ~ WaveOne Gold (p > 0.05).

A statistically significant interaction was detected between irrigant type and file system for mean torque values (p = 0.002), indicating that torque behavior varied depending on the specific irrigant–file combination used. However, no significant interaction effect was observed for the maximum torque (p = 0.097) or preparation time (p = 0.067) (Table 4). When descriptive trends were examined across all irrigants, T-Endo MUST consistently produced the highest mean and maximum torque values, whereas WaveOne Gold generated the lowest measurements; Reciproc Blue exhibited intermediate behavior. The highest maximum torque value in the dataset was recorded with T-Endo MUST in saline. Overall, irrigant type did not significantly influence torque values or preparation time, whereas file system selection significantly affected torque generation. The mean torque behavior was dependent on the irrigant–file combination, whereas the preparation time remained stable regardless of the tested variables.

**Table 4:** Two-Way ANOVA (3 Irrigants × 3 File Systems).

	Preparation Time		Mean Torque		Maximum Torque	
Factor	F	p	F	p	F	p
File System	1.659	.197	15.274	.001	6.986	.001
Irrigant	1.212	.303	0.532	.593	0.673	.512
File × Irrigant	2.285	.067	5.032	.002	2.132	.097
Adjusted r²	.072		.322		.185	

*Note. Preparation Time: No significant effects were observed. For the Mean Torque: Significant interaction effect was present. Maximum Torque: No significant interaction effect.

Because a significant interaction between file system and irrigant type was detected for mean torque (p = 0.002), descriptive comparisons of the nine file–irrigant combinations were performed to explore the direction of this interaction. The highest mean torque value was recorded for T-Endo MUST in saline, whereas the lowest value was observed for WaveOne Gold in saline. Within the Reciproc Blue groups, the highest mean torque was observed with NaOCl. WaveOne Gold exhibited its highest torque values in the EDTA group, while T-Endo MUST showed the highest torque values in saline. These findings indicate that the effect of irrigant type on torque depended on the specific file system used (Table 5).

**Table 5:** Mean torque values according to file–irrigant combinations.

File System	Saline	Sodium hypochlorite	EDTA
Reciproc Blue	1.52 ± 0.45	1.96 ± 0.46	1.95 ± 0.40
WaveOne Gold	1.54 ± 0.31	1.76 ± 0.21	1.81 ± 0.29
T-Endo MUST	2.52 ± 0.43	2.04 ± 0.45	2.13 ± 0.42

## Discussion

NiTi endodontic instruments can experience stress concentrations during operation, and these stresses may contribute to mechanical complications depending on the structural and material characteristics of the instrument [17]. Over recent decades, substantial advancements in NiTi metallurgy, thermomechanical processing, and heat-treatment protocols have markedly improved the flexibility, transformation behavior, and overall mechanical performance of alloys [18–20]. Consequently, understanding how modern heat-treated NiTi instruments respond to dynamic mechanical stresses is essential for evaluating their operational reliability.

Reciproc Blue, WaveOne Gold, and T-Endo MUST incorporate distinct heat treatments and cross-sectional geometries that influence their flexibility, metal mass distribution, and stress response during reciprocating motion [21–23]. In the present study, these instruments were examined under real-time torque monitoring to characterize their behavior under dynamic loading conditions. Although thermomechanically processed NiTi alloys demonstrate enhanced flexibility, abrupt torque elevations can still result in localized stress concentrations, which may contribute to mechanical complications when the apical portion of the instrument binds while the shank continues to rotate [24].

Torque generation in NiTi instruments is influenced by multiple material- and design-dependent factors, including the cross-sectional geometry, taper, alloy flexibility, reciprocating kinematics, and lubrication at the file–dentin interface [25–27]. Traditional static torsional and cyclic fatigue tests do not adequately reflect the dynamic mechanical behavior of reciprocating instruments, as they evaluate performance under constant, unidirectional loading conditions that differ from the alternating movements experienced during operation [28^,^^29^]. As reciprocating instruments are not intended to rotate continuously in a single direction, static test outcomes may poorly represent their mechanical response under actual motion patterns [13]. For this reason, dynamic torque analysis has gained prominence as a method capable of capturing real-time stress fluctuations within alloys during instrumentation [30]. In this study, the X-Smart IQ system was employed to quantify torque fluctuations during canal shaping at short time intervals, allowing a more realistic assessment of file behavior.

### Effect of irrigating solutions

NaOCl acts as a potent oxidizing medium capable of degrading organic components at the dentin–instrument interface, whereas EDTA functions as a chelating agent that targets inorganic constituents and alters the surface characteristics relevant to mechanical contact [31,32]. Despite extensive research on their physicochemical effects on dentin and smear layer integrity, relatively few studies have evaluated their influence on the operative torque during instrumentation [14].

In the current study, saline, NaOCl, and EDTA did not result in statistically significant differences in mean torque, maximum torque, or preparation time. These results are consistent with reports indicating that even surfactant-modified NaOCl exerts a minimal influence on torque behavior under similar experimental conditions [13]. In contrast, another study observed a reduction in torque generation when EDTA was used in mandibular premolars [14], suggesting that discrepancies across studies may stem from variations in instrument design, substrate type (resin blocks versus natural dentin), and irrigation protocols. Overall, the influence of chemical media on torque appears to be multifactorial and strongly dependent on file-specific mechanical and geometric characteristics.

### Effect of file systems

Significant differences in mean and maximum torque values were observed among the evaluated file systems. In particular, Reciproc Blue exhibited slightly higher torque values than WaveOne Gold, consistent with a previous report [9]. This behavior may be associated with Reciproc Blue’s larger taper and S-shaped cross-section, which increases its core metal mass and degree of engagement with canal walls [33]. Laboratory evidence indicates that Reciproc Blue exhibits enhanced cyclic fatigue resistance, a property attributed to its proprietary heat-treatment protocol [34]; however, the increased flexibility associated with such thermal processing may also reduce torsional resistance [35]. These relationships suggest that the maximum torque reflects the intrinsic torsional strength of a system rather than its fatigue characteristics.

WaveOne Gold consistently produced the lowest torque values, likely because of its reduced metal mass and parallelogram cross-section, both of which limit dentin engagement and decrease mechanical loading [36]. In the present study, the T-Endo MUST generated the highest mean and maximum torque values. Although comparative data remain unavailable for this instrument, its S-shaped geometry and cutting efficiency may increase frictional interaction and load transmission during reciprocating motion [37]. The current findings provide baseline mechanical data that may support future material-based investigations of this file system. The higher mean and peak torque values observed with the T-Endo MUST system may be associated with its geometric design characteristics. Instruments with greater core mass and more pronounced cutting blades tend to engage dentin more aggressively, which can increase torsional load during reciprocating instrumentation [8^,^^25^]. In addition, cross-sectional geometry and metal mass distribution influence the contact area between the file and canal walls, thereby affecting frictional resistance and torque demand [27]. Therefore, the elevated torque values observed in the present study should not be interpreted solely as an indicator of superior cutting efficiency; they may also reflect increased frictional interaction and a higher likelihood of taper-lock during canal preparation, particularly in oval canals [37]. These interpretations should be considered associative rather than causal, as the present experimental design does not allow direct determination of the specific structural factors responsible for the observed torque behavior.

Although Reciproc Blue has been reported to exhibit an S-shaped or S-like cross-sectional design [33], the higher torque values observed with T-Endo MUST suggest that additional geometric factors beyond general cross-sectional classification may be involved. Differences in core diameter, taper progression, and cutting blade configuration may influence the degree of dentin engagement during instrumentation. Instruments with greater effective metal mass or more aggressive cutting blades may establish increased contact with canal walls, thereby elevating frictional resistance and torque generation [25,27]. This may partly explain the higher torque values observed in the T-Endo MUST system compared with the other instruments evaluated in the present study.

In contrast, WaveOne Gold, with its offset parallelogram cross-sectional design, may reduce the extent of continuous contact with dentin, which could contribute to its lower torque values observed in the present study [36]. The present findings indicate that cross-sectional classification alone may be insufficient to predict mechanical behavior, and that subtle differences in internal geometry and mass distribution can significantly influence torque generation.

### Preparation time

The preparation time is influenced by factors such as canal geometry, instrument design, alloy flexibility, and kinematics, all of which affect frictional resistance and material–dentin interaction during shaping [38^,^^39^]. In previous investigations, one study reported that reciprocating motion enabled faster preparation than continuous rotation [38], whereas another study found comparable shaping durations depending on the instrument design and experimental conditions [39]. In the present study, preparation times did not differ significantly among the systems, although WaveOne Gold exhibited the shortest duration and T-Endo MUST the longest. This trend may reflect variations in cutting efficiency, taper, and frictional contact arising from differences in the instrument geometry and core metal mass.

A significant interaction was detected between the file system and irrigant type for the mean torque, indicating that torque behavior arises from a combined chemical–mechanical interaction rather than being governed by a single independent factor. No interaction was observed for the maximum torque or preparation time, supporting the concept that torque spikes are more strongly influenced by instrument geometry and localized binding conditions than by short-term lubrication effects.

The significant interaction observed between file system and irrigant type for mean torque suggests that torque generation is influenced by the combined effect of instrument design and the surrounding chemical environment. In the present study, T-Endo MUST exhibited the highest torque values particularly in saline, which may reflect conditions in which mechanical factors predominate in the absence of significant chemical alteration of dentin surfaces [31^,^^32^^].^

In contrast, NaOCl and EDTA may alter dentin surface properties and the physicochemical conditions at the file–dentin interface [31,32]. Previous studies have shown that these irrigants can influence the mechanical behavior of NiTi instruments and modify surface characteristics, although their overall effect remains inconsistent in the literature [13,14]. These chemical interactions may affect lubrication conditions and frictional resistance during instrumentation [8,14]. For example, Reciproc Blue demonstrated relatively higher torque values in NaOCl, which may reflect differences in how its cross-sectional design interacts with chemically modified dentin surfaces.

These findings suggest that the influence of irrigating solutions on torque is instrument-dependent, and interpreting only main effects without considering interaction effects may oversimplify the underlying mechanical behavior [8]. This interaction further indicates that torque behavior cannot be generalized across different irrigation conditions, and that the mechanical response of a given instrument may vary depending on the chemical environment present during canal preparation.

Torque plays a central role in stress accumulation within NiTi instruments and is directly associated with the risk of structural failure under applied loads [40]. Real-time torque monitoring provides a means to quantify these stress fluctuations as they occur, offering more accurate insights into the mechanical performance of a file during dynamic operation [14^,^^30^]. As torque generation is an inherent outcome of reciprocating motion, understanding how instrument design, alloy flexibility, and cross-sectional geometry influence stress transmission is essential for characterizing the material behavior of heat-treated NiTi systems.

Because variability was relatively high in some groups, a post-hoc power analysis was performed focusing on the irrigant comparison. Based on the observed mean torque differences among irrigants (Table 2), the estimated effect size was very small (Cohen’s f = 0.087). Accordingly, the achieved power to detect such a small effect with N = 90 at α = 0.05 was limited (≈0.10), indicating that very small irrigant-related differences may remain undetected. However, the observed differences among irrigants were minimal under the present protocol, suggesting that any irrigant effect on mean torque—if present—is likely small in magnitude. A sensitivity analysis further showed that the present sample size would provide 80% power to detect effects of approximately f = 0.33 (or larger).

This study has several limitations related to the material and experimental considerations. Only mandibular premolars with oval-shaped canals were evaluated, restricting the applicability of the findings to other anatomical geometries that may impose different mechanical loading conditions. The moderate curvature (<30°) represented in the sample may not fully reproduce the stress distributions in more severely curved canals. Moreover, only a single file size per system was tested, even though variations in tip size and taper alter the metal mass and may influence torque demand. Although instrumentation was performed by an experienced operator to maintain consistency, operator-related factors could not be eliminated. Future investigations should incorporate specimens with greater geometric variability, integrate high-resolution methods such as micro-CT to evaluate material removal patterns, and include a broader range of thermomechanically treated NiTi systems to improve the comparative assessment of material–environment interactions. Another limitation of the present study relates to the irrigation protocol. Conventional needle irrigation was used without sonic or ultrasonic activation. In oval canals, the well-known vapor lock phenomenon may limit irrigant exchange in the apical third of the canal, preventing effective penetration of the solution to the file–dentin interface [1,2]. Consequently, the potential lubricating and debris-removal effects of irrigants such as EDTA or NaOCl may not have been fully expressed during instrumentation [31]. This limitation may partially explain the absence of significant differences among irrigation solutions in the present study. Future investigations incorporating activated irrigation systems may provide a more comprehensive understanding of how irrigant dynamics influence torque behavior during root canal preparation. Real-time torque measurements should be interpreted as a mechanical proxy of stress generation during instrumentation and should not be considered a direct predictor of clinical instrument fracture.

Torque accumulation during root canal preparation is an important contributor to instrument separation and dentinal stress. This study demonstrated that the choice of irrigating solution does not significantly influence operative torque when using reciprocating NiTi systems, indicating that irrigant selection may therefore be guided primarily by antimicrobial and smear layer management objectives within the limitations of the present experimental model. In contrast, the type of file system used had a notable impact on torque generation, underscoring the importance of instrument design in reducing torsional stress and maintaining procedural safety in clinical practice.

## Conclusions

Within the limitations of this in vitro study, irrigant type did not significantly influence torque; nevertheless, a significant interaction between file system and irrigant type was observed for mean torque, indicating that torque behavior depended on the specific file–irrigant combination. These findings demonstrate that torque generation is influenced by an interaction-based effect between instrument design and the surrounding chemical environment, rather than by either factor independently. However, these findings should not be interpreted as evidence that irrigation has no mechanical relevance during instrumentation. In clinical situations involving complex canal anatomies or severe curvatures, the interaction between irrigant properties and mechanical instrumentation may differ. Therefore, irrigant selection should consider both antimicrobial effectiveness and potential mechanical influences during root canal preparation. Overall, the present findings highlight the importance of evaluating torque behavior under combined mechanical and chemical conditions when assessing the performance of reciprocating NiTi systems.

## Supporting information

S1 Data Raw dataset of torque values and preparation times for all experimental groups (Excel file). (XLSX)

S1 Fig Graphical abstract illustrating the experimental design and workflow of the study, including sample selection, grouping based on irrigating solutions and reciprocating file systems, and real-time torque recording during root canal preparation. (JPEG)
