# Supplementary figures and images for: Real-time torque behavior of reciprocating nickel–titanium instruments using different irrigating solutions
Source: PLoS One. 2026 Apr 17;21(4):e0347424. doi: 10.1371/journal.pone.0347424 (PMC13089759; doi:10.1371/journal.pone.0347424)

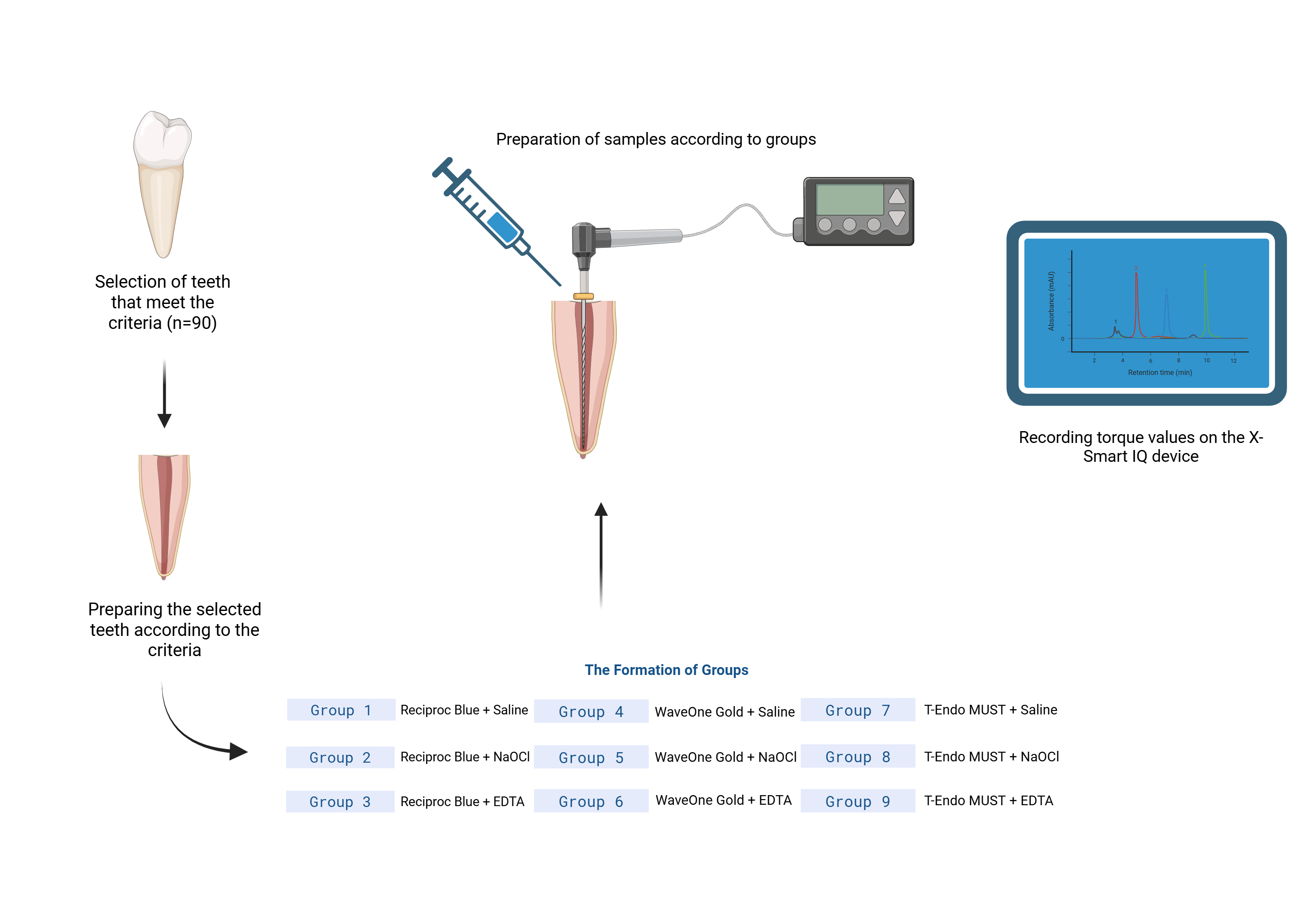

Supplement: S1 Fig — (JPEG) [file pone.0347424.s002.jpeg]
